# Suspended detrital particles support a distinct microbial ecosystem in Palmer Canyon, Antarctica, a coastal biological hotspot

**DOI:** 10.1007/s00300-025-03380-y

**Published:** 2025-04-07

**Authors:** Elizabeth Connors, Katherine L. Gallagher, Avishek Dutta, Matthew Oliver, Jeff S. Bowman

**Affiliations:** 1 Scripps Institution of Oceanography, UC San Diego, 8622 Kennel Way, La Jolla, CA 92037 USA; 2 Scripps Polar Center, UC San Diego, La Jolla, CA USA; 3 School of Marine and Atmospheric Sciences, Stony Brook University, Stony Brook, NY USA; 4 Department of Geology, University of Georgia, Athens, GA USA; 5 Savannah River Ecology Laboratory, University of Georgia, Aiken, SC USA; 6 School of Marine Science and Policy, University of Delaware, Newark, DE USA

**Keywords:** Microbial ecology, Suspended particles

## Abstract

The coastal region of the Western Antarctic Peninsula is considered a biological hotspot with high levels of phytoplankton productivity and krill biomass. Recent in situ observations and particle modeling studies of Palmer Canyon, a deep bathymetric feature in the region, demonstrated the presence of a recirculating eddy that traps particles, retaining a distinct particle layer over the summer season. We applied metagenomic sequencing and Imaging Flow Cytobot (IFCB) analysis to characterize the microbial community in the particle layer. We sampled across the upper water column (< 200 m) along a transect to identify the locations of increased particle density, categorizing particles into either living cells or cellular detritus via IFCB. An indicator species analysis of community composition demonstrated the diatom *Corethron* and the bacteria *Sulfitobacter* were significantly highly abundant in samples with high levels of living cells, while the mixotrophic dinoflagellate *Prorocentrum texanum* and prokaryotes Methanomassiliicoccales and *Fluviicola taffensis* were significantly more abundant in samples with high detritus within the particle layer. From our metagenomic analysis, the significantly differentially abundant metabolic pathway genes in the particle layer of Palmer Canyon included pathways for anaerobic metabolism, such as methanogenesis and sulfate reduction. Overall, our results indicate that distinct microbial species and metabolic pathway genes are present in the retained particle layer of Palmer Canyon.

**Supplementary Information:**

The online version contains supplementary material available at 10.1007/s00300-025-03380-y.

## Introduction

In marine environments, particulate organic matter hosts distinct microbial communities compared to the surrounding seawater, especially with depth (Jain et al. 2021). Mid-water suspended particles constitute the majority of marine particulate organic carbon in the ocean and support most of the microbial carbon respiration in the mesopelagic ocean (Baltar et al. 2010). These subsurface organic particles and the microorganisms that degrade them play a central role in controlling the transport, cycling, and stocks of nutrients including carbon, nitrogen, and phosphorus (Baumas and Bizic 2024). The prevalence of anaerobic processes in particle-associated nutrient cycling, including sulfate reduction and methanogenesis, has also been recently discovered in the reduced oxygen micro-niches on marine particles (Ditchfield et al. 2012; Riemann et al. 2022; Siebers et al. 2024).

A better understanding of which microbial species and metabolic strategies are present on suspended particles is necessary, as metabolic activity on suspended particles can weaken the efficiency of the biological carbon pump (Duret et al. 2019). This vertical transfer of phytoplankton-derived organic matter to the deep ocean ranges from 5 to > 12 Pg C yr^−1^ and is attenuated with depth in the water column (Siegel et al. 2016). The microbial species and metabolic strategies of rapidly sinking particles important to carbon flux have been described previously (Poff et al. 2021). However, the prokaryotic species and metabolic strategies present on the majority of marine particles, which are suspended in the water column below the surface ocean, remain relatively unclear (Fadeev et al. 2021). Of particular interest is the presence of metabolically active bacteria in reduced oxygen micro-niches that can form inside particles, as oxygen consumption has been used as a proxy for total bacterial respiration rates in sinking particles in the past (McDonnell et al. 2015; Belcher et al. 2016).

A recent discovery of a subsurface recirculating eddy and its retention of detrital material near Palmer Station, Antarctica, provides an opportunity to understand the microbial composition and potential metabolisms associated with high detrital particle densities in the polar seas. Along the western Antarctic Peninsula, there are deep submarine canyons near many penguin breeding colonies and their marine feeding grounds. These canyons are known biological hotspots with high abundances of phytoplankton and krill (Schofield et al. 2013). The increased biological activity of these sites was previously attributed to the upwelling of warm Upper Circumpolar Deep water in the canyons (Schofield et al. 2013; Kavanaugh et al. 2015). However, in a recent lab experiment, upwelling deeper waters did not stimulate phytoplankton growth (Carvalho et al. 2019). Furthermore, upwelling into the surface mixed layer was not observed over the canyon (Hudson et al 2019). Further modeling and in situ observations indicated the presence of a recirculating eddy in Palmer Canyon, which may sustain the high level of phytoplankton and krill biomass present near Anvers Island, Antarctica (Hudson et al. 2021, 2022). This subsurface recirculating eddy both retains living phytoplankton cells and detrital particles at relatively shallow (< 150 m) depths over Palmer Canyon and may even increase zooplankton residence times in the canyon (Hudson et al. 2019).

Here, we first identify the Palmer Canyon detrital particle layer using images of particles from an Imaging Flow Cytobot (IFCB). The IFCB classified events into either living cells or detritus and counted the density of both particles from our sampling profiles moving into the canyon. We then leveraged cell abundances and amplicon sequencing (16S rRNA gene and 18S rRNA gene sequencing) to contrast the microbial community present where living cells had the highest abundance at 5 m, to community composition in samples from the high-density detrital particle layer at 75 m in Palmer Canyon. Finally, we divided our samples into two categories (those with low or high levels of detritus) and determined the differential relative abundance of microbial metabolic pathway genes (from shotgun metagenomic sequencing) present in the high detritus samples, including especially anaerobic metabolism. We designed our study to improve our understanding of diversity and potential adaptation strategies of the microbial communities in a less explored habitat in the Antarctic environment.

## Materials and methods

**Fig. 1:**
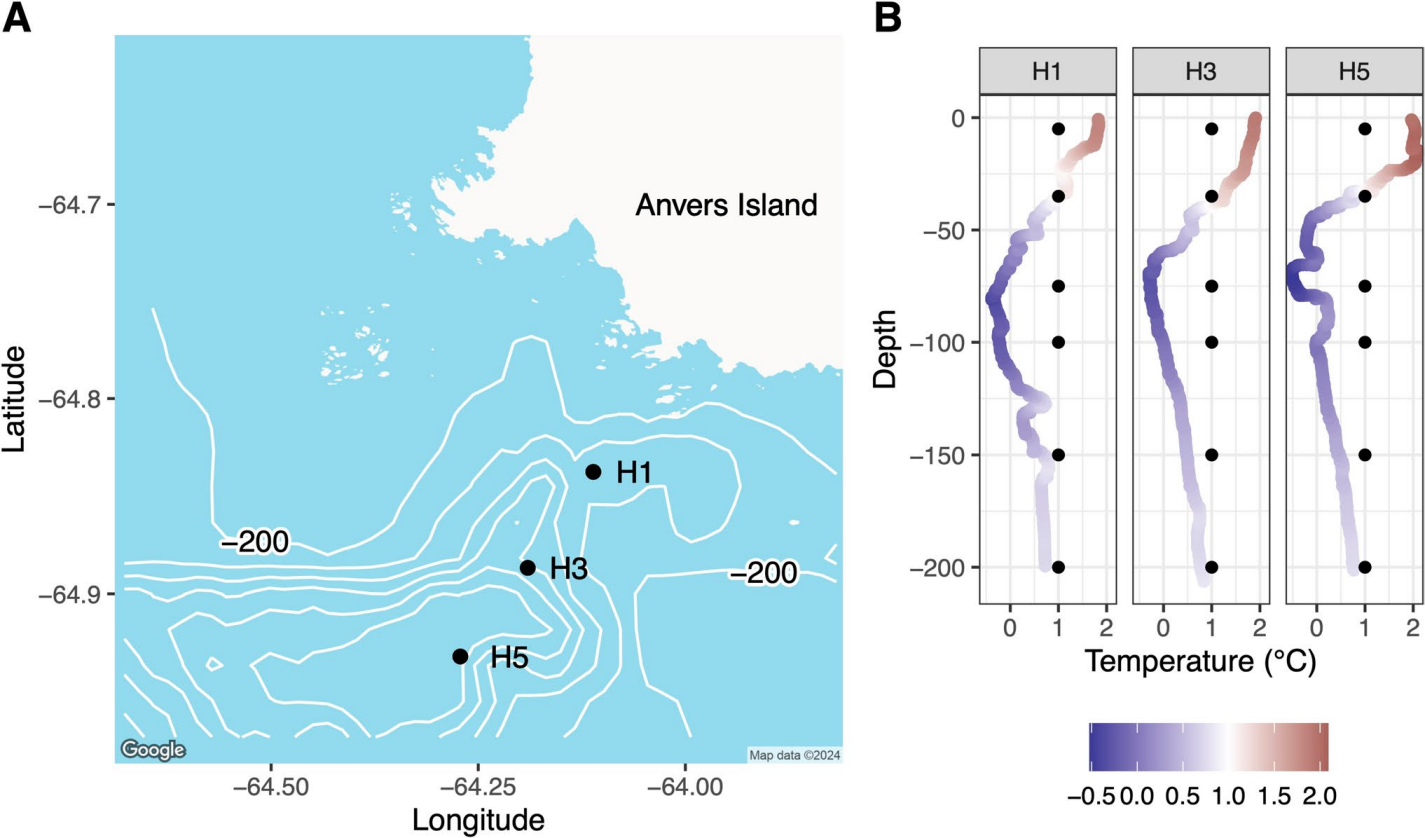
**A** Location and bathymetry of stations sampled in Palmer Deep Canyon, near Anvers Island, Antarctica with bathymetry lines every 200 m, where the bottom depth of H1 (450 m) is shallower than H3 (800 m) and H5 (1250 m). **B** CTD temperature profile of the three stations, with water collection depths marked with points

A CTD (Seabird SBE19v2) was deployed from the small research vessel *Hadar* on 7 March 2020, at three stations: H1 (Fig. 1A, 64.8375°S, 64.1111°W), H3 (− 64.88669, − 64.19028), and H5 (− 64.93207, − 64.27167) to measure physical oceanographic conditions (temperature, salinity), beam transmission (via a WET lab C-Star Transmissometer), and to collect seawater samples for downstream analysis. Seawater was collected from an integrated carousel water sampler with Niskin bottles on the CTD from 6 depths (5, 35, 75, 100, 150, and 200 m, Fig. 1B) at each station. Collected seawater was divided into samples for IFCB (McLane Labs, Falmouth, MA, USA) analysis, triplicate flow cytometry samples, and triplicate DNA samples, which were then extracted and used both for amplicon and shotgun metagenomic sequencing analysis.

### Imaging flow cytobot

**Fig. 2:**
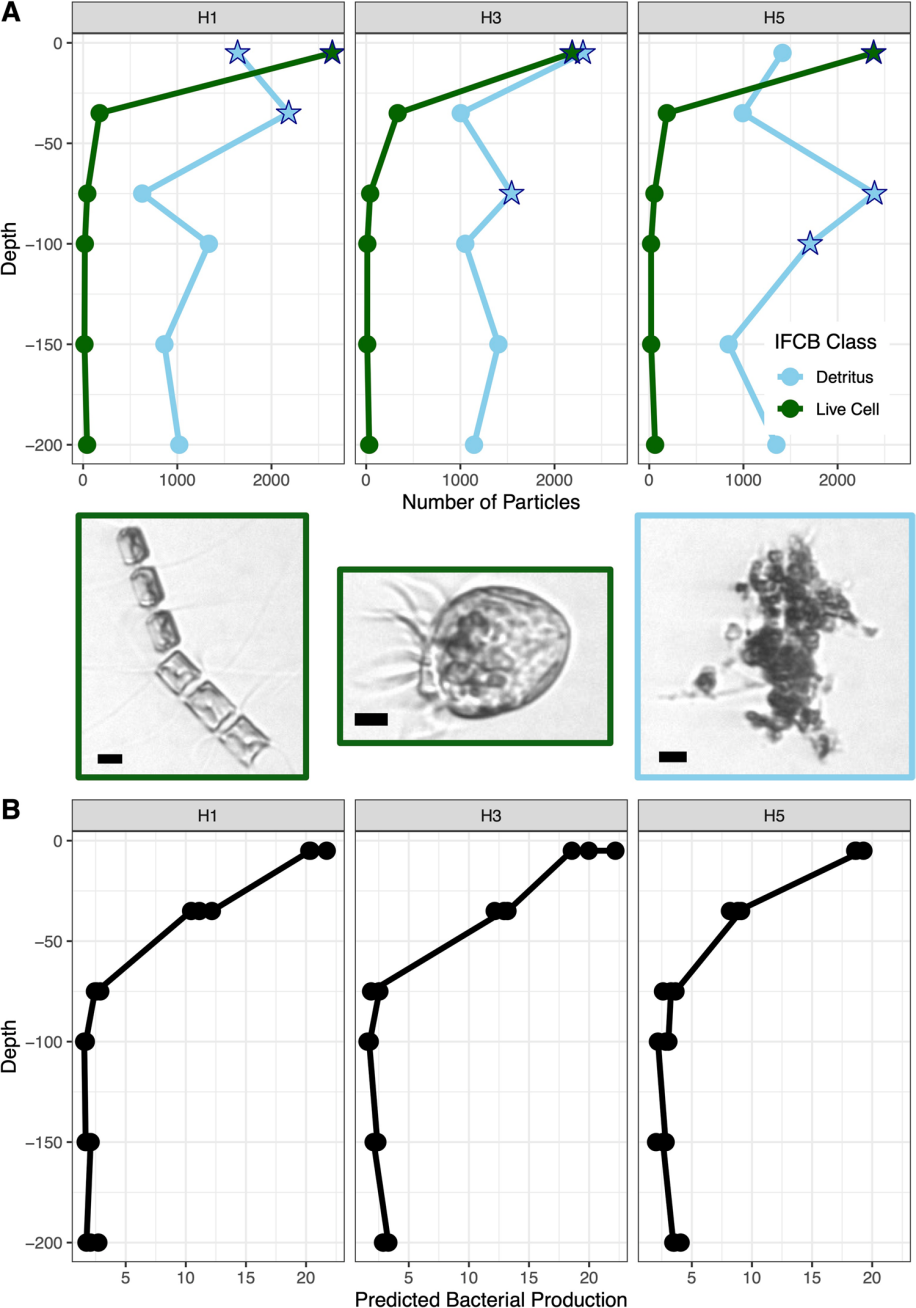
**A** Imaging Flow CytoBot (IFCB) image analysis over the three stations (H1, H3, and H5) and the depths sampled. IFCB images are classified as detritus or live cells and high levels of either exceeded 1500 images are marked with stars. Center is example images of both (two live cell and then a detritus image) with scale bars of 5 µm on each. **B** Predicted bacterial production (pmol leucine L^−1^ h^−1^) over the three stations and the depths sampled

The IFCB samples were utilized to identify the subsurface particle layer and categorize identified particles into either live cells or detritus (Hudson et al. 2021). The samples for IFCB were collected in 50-mL Falcon tubes and remained dark and cold until processed at Palmer Station, Antarctica. On the IFCB instrument, images of all particles in a 5 mL seawater subsample were collected with scattering (PMTA) and chlorophyll (PMTB) sensors (Olson and Sosik 2007). Feature extraction on the images was performed following the Palmer Long Term Ecological research program protocol (Nardelli et al. 2022) using the MATLAB IFCB Toolbox (https://github.com/hsosik/ifcb-analysis/wiki). Images with PMTB greater than 0.01 (~ 77% quantile) were classified as live cells for this study, as discussed in Hudson et al. (2021), where these IFCB data were originally published. For our analysis, any sample where greater than 1500 IFCB images of live cells or detritus were observed by the IFCB in 5 mL was labeled as “high” in live cells or detritus, respectively (stars in Fig. 2A).

### Flow cytometry

Flow cytometry samples were utilized to count phytoplankton and bacterial cell abundances. These samples were prefiltered to avoid clogging the instrument (40 µm) and aliquoted into 2-mL sample tubes and then run on a AccuriC6 flow cytometer (BD Biosciences, Franklin Lakes, NJ, USA) which interrogates cells with a blue laser (488 nm). All samples were run unstained (for autofluorescence, AF) and stained and incubated in the dark for 15 min with the nucleotide stain SYBR Green 1 (Molecular Probes, Inc.) diluted 2000× (SYBR). Quality control for total cell counts was confirmed by adding 10 µL of 1:2500 diluted 1 µm Fluoresbrite Yellow Microspheres (Polyscience Inc.) to each sample. All SYBR samples were run on “slow” with a flow rate of 13 µL min^−1^ for one minute and measured for forward scatter, side scatter, and green emission (488/533 nm excitation/emission). All AF samples were run on “fast” with a flow rate of 66 µL min^−1^ for three minutes and measured for forward scatter, side scatter, and both red (488/675 nm excitation/emission) and yellow emission (488/585 nm excitation/emission).

For SYBR samples, populations were identified using a self-organizing map (SOM) from forward scatter, side scatter, and green emission following previous methods (Bowman et al. 2017; Wilson et al. 2021), for AF samples, the same methods were used on forward scatter, side scatter, and both red and yellow emission. In brief, five randomized samples were selected to construct a training set. These data were then trained using a toroidal map with a grid size of 41 × 41 with the ‘kohonen’ package in R (Wehrens and Kruisselbrink 2018). Populations were identified using k-means clustering and *k* = 3 for SYBR samples and *k* = 4 for AF samples, which was then used to classify events in all flow cytometry samples (Supplemental Fig. 1A).

Three clusters were identified as high chlorophyll (high chl), low chlorophyll (low chl), and high phycoerythrin (high PE) in the AF model; two clusters were identified as high nucleic acid (HNA) and low nucleic acid (LNA) clusters in the SYBR model (Supplemental Fig. 1B). These clusters were converted into cells mL^−1^ from events µL^−1^ volume run and then combined to form a total cell count (either auto fluorescent (AF) or bacterial (SYBR) abundance in cells mL^−1^) for each sample. Total cell count outliers (2 observations) were removed when their values were outside the range Q1 − 1.5 × (Q3 − Q1), Q3 + 1.5 × (Q3 − Q1), where Q1 and Q3 are the first and third quartiles, respectively.

### DNA extraction and amplicon sequences

DNA samples were utilized to determine the microbial community composition (via 16S and 18S rRNA gene sequencing) and metabolic genes (via shotgun metagenomic sequencing). For every DNA sample, 1 L of seawater was filtered through a sterile 0.2 µm Supor membrane disk filter (Pall Corporation, Port Washington, NY, USA) and stored at − 80 °C until extraction. Filters were extracted using the KingFisher™ Flex Purification System and MagMax Microbiome Ultra Nucleic Acid Extraction kit (ThermoFisher Scientific, Waltham, Massachusetts, USA). For amplicon sequencing, extracted DNA was sent to Argonne National Laboratory for amplicon library preparation and sequencing and sequencing using the Illumina MiSeq platform with the primers 515F and 806R for 16S rRNA sequencing (Walters et al. 2016), 1380F and 1505R for 18S rRNA sequencing (Amaral-Zettler et al. 2009) in a 2 × 151 bp library architecture. All amplicon sequences were submitted to NCBI SRA under BioProject PRJNA901488.

Illumina reads were filtered, denoised, and merged with DADA2 (Callahan et al. 2016) and then analyzed with paprica v0.7.1 (Bowman and Ducklow 2015). Paprica utilizes phylogenetic placement with Gappa (Czech et al. 2020) EPA-ng (Barbera et al. 2019) and Infernal (Nawrocki and Eddy 2013), and RefSeq to place query reads on a reference tree constructed from the full-length 16S rRNA genes from all completed genomes in GenBank (Haft et al. 2018) or 18S rRNA genes from all completed genomes in PR2 4.13.0 (Guillou et al. 2013). All unique reads were assigned to internal branches or terminal branches on the reference tree. Once assigned, unique reads that were assigned as metazoan mitochondria or chloroplasts were omitted, as well as any reads that only appeared once.

For our beta diversity analysis of either the 16S rRNA or 18S rRNA gene amplicon sequences, unique reads were first cumulative sum scaled (CSS) to normalize for sampling depth across samples with the R package metagenomeSeq (Paulson et al. 2013). Then, non-metric multidimensional scaling (NMDS) of Bray–Curtis distance and data dispersion (via the betadisper function) of the CSS-scaled relative abundance table and a calculation for the Shannon diversity index from the unscaled relative abundances were conducted with the vegan package (Oksanen et al. 2022). Post hoc analysis of variance in Bray–Curtis distances across station, depth, IFCB live cell count, and IFCB detritus count was conducted with the R package pairwiseAdonis (Martinez Arbizu 2020).

We conducted four indicator species analyses with the R package indicspecies (De Caceres and Legendre 2009) to determine the indicator species in high IFCB live cell count or high IFCB detritus count conditions from the relative abundance of the 18S amplicon sequences or from the relative abundance of the 16S amplicon sequences, respectively. Because depth was a significant confounding variable, both IFCB live cell indicator species analyses were only run on the shallowest depths (5, 35 and 75 m), while both IFCB detritus indicator species analyses were run on only the mid-water depths where the highest level of detritus was found at specific stations (35, 75 and 100 m). For all indicator species analyses, we only reported statistics for genera that have a *p*-value of < 0.05 for high levels of either live cells or detritus (Supplementary Table 1). Heatmaps of these data were created with the package pheatmap (Kolde 2019). Finally, as the 16S amplicon data in this study were part of a larger study that predicted bacterial production from community composition (Connors et al. 2024), we report the predicted bacterial production of these samples from the Palmer-specific model in that study to compare bacterial carbon uptake across the stations and with depth.

### Metagenomic sequencing and differential abundance analysis

A subset (*n* = 21) of the DNA samples (total amplicon *n* = 54) was sequenced for shotgun metagenomes to compare metabolic pathway genes across the two levels of IFCB detritus (high vs. low) depths with the highest values for IFCB detritus (5 m, 35 m and 75 m). Metagenomic sequencing was performed on the Illumina NovaSeq platform at the UC San Diego Microbiome Core. Raw reads were quality controlled, assembled, and binned with the iMAGine pipeline (Dutta et al. 2023). iMAGine includes the dependencies fastp (Chen et al. 2018) for filtering, metaSPAdes (Nurk et al. 2017) for assembling the reads, QUAST (Gurevich et al. 2013) for analyzing the assembly quality, BWA-MEM (v0.7.17) for aligning the raw reads to the assembly (Li 2013), samtools for modifying alignment files (Li et al. 2009), metabat2 (Kang et al. 2019) for binning contigs, and checkM (Parks et al. 2015) for quality assessment of the bins. High-quality bins (completeness higher than 70% and contamination lower than 5%) were combined with the bins of Dutta et al. (2023) and dereplicated. The bins were used to construct a database for Diamond blastx (Buchfink et al. 2015).

Metagenomes reads were searched against the database, and the results tallied using custom scripts to create an abundance table by bin and gene. Functional annotations of the genes were annotated with eggNOG DB v5.0.2 (Huerta-Cepas et al. 2019). The package deseq2 (Love et al. 2014) was then used to determine the differential abundance of unscaled counts of metagenome reads at each depth that were sequenced with sufficient replicates (*n* = 16, Fig. 5A). As deseq2 analysis requires binary sample categorization (i.e., x vs. y), we compared high IFCB detritus to low IFCB detritus samples and reported the positive log change (genes that have significantly higher abundance in high detritus samples).

Finally, to investigate if metagenomic signatures of methanogenesis and sulfate reduction were more dominant in Palmer Deep Canyon at 75 m, we computed the normalized gene abundance of a sample by dividing the relative abundance of each gene by the relative abundance of the single copy marker gene rpoB (K03043). We then combined, via addition, the normalized abundance of every significantly differentially abundant metagenomic read that mapped to KEGG modules for low-oxygen metabolism including methanogenesis (M00357 and M00563), sulfate reduction (M00176 and M00596), and denitrification (M00529) to directly compare metabolic signature across the stations.

## Results 

### Counting live cells and detritus moving into Palmer Deep Canyon

The live cell and detritus particles imaged by the IFCB showed different patterns with depth. IFCB live cell images decreased significantly with depth (green line in Fig. 2A, ANOVA *p*-value: 8.8 × 10^−16^ for 5 m vs. other depths) from their peak at 5 m, where all stations had greater than 2000 live cell images captured from 5 mL of water. However, the subsurface depths where significantly higher levels of detritus were found (> 1500 images of detritus taken by the IFCB from 5 mL of seawater, ANOVA *p*-value: 2.0 × 10^−5^) occurred at deeper depths across the stations moving into Palmer Deep Canyon (deepest is 35 m at H1 to 100 m at H5, blue stars in Fig. 2A). Both total IFCB images for live cell and detritus were not significantly different across stations (ANOVA *p*-value: 0.99 for IFCB live cell images and *p*-value: 0.848 for IFCB detritus images, Fig. 2A).

Most of the other parameters measured followed a similar pattern to live cell images, with significant changes with depth. Beam transmission increased from 70 to 90% and salinity increased from 32.5 PSU to 33.5 PSU with depth in the upper 50 m for all stations. Predicted bacterial production decreased significantly with depth from 20 pmol leucine L^−1^ h^−1^ at the surface to below 5 pmol leucine L^−1^ h^−1^ below 40 m (Fig. 2B, ANOVA *p*-value: 4.7 × 10^−11^ across depths). Both autofluorescent and SYBR flow cytometry cell counts were significantly higher at 5 m (which was where IFCB live cell images were significantly higher), with total cell counts at ~ 7000 cells mL^−1^ for AF and ~ 500,000 cells mL^−1^ for SYBR (Supplementary Fig. 1, AF and SYBR ANOVA *p*-value: 2.0 × 10^−16^). Autofluorescent cell counts were not significantly different across stations (ANOVA *p*-value: 0.75) or across IFCB detritus levels in the subsurface (below 5 m high vs. low ANOVA *p*-value: 0.19). SYBR total cell counts, however, did show differences across stations, with significantly higher total cells at H3 at 75 m (below 5 m which had high IFCB detritus, ANOVA *p*-value: 9.0 × 10^−3^), and significantly higher cell counts at H1 beneath 100 m (Supplementary Fig. 1B, ANOVA *p*-value: 3.2 × 10^−6^).

### Microbial community composition and indicator species

**Fig. 3:**
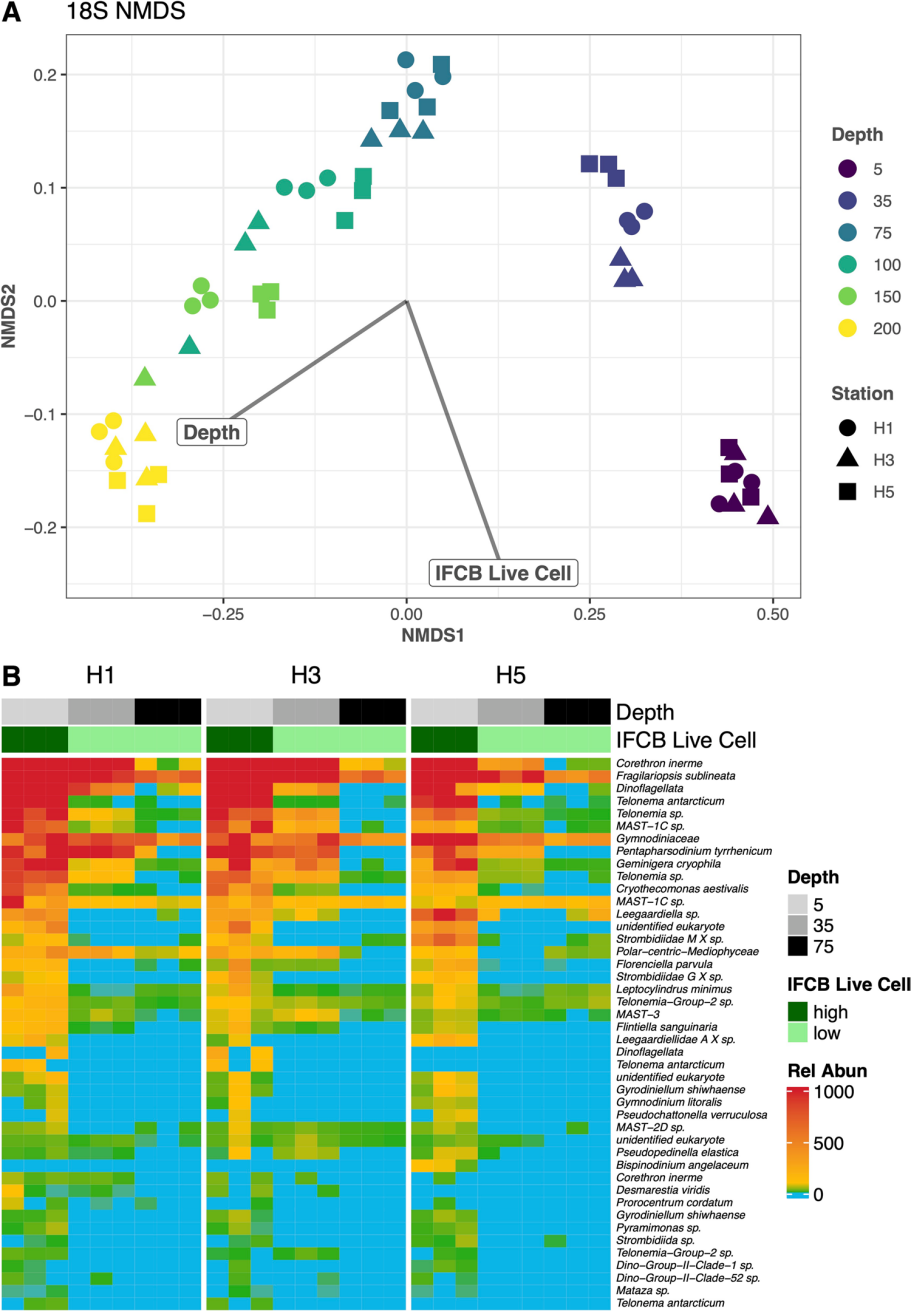
Relative abundance of 18S rRNA gene amplicon sequences across the transect **A** is an NMDS plot (stress = 0.04) where the shape of point is Station (H1, H3 and H5) and color is Depth. IFCB Live Cells and Depth were significant (ADONIS *p* = 0.001) while IFCB Detritus and Station were not significant (ADONIS 0.072 and 0.159, respectively) for the NMDS. **B** Relative abundances of the significant (*p* < 0.05) indicator species for high (> 1500) IFCB Live Cell images at 5, 35 and 75 m depth

The most dominant genera in the eukaryotic community compositions (relative abundance of 18S rRNA sequences) were the diatom genera *Corethron* and *Fragilariopsis* across all three stations in shallow water (5 and 35 m). In addition to diatoms, dinoflagellates such as *Gymnodiniaceae*, flagellates such as *Telonema,* and cryptophytes such as *Geminigera* were also highly relatively abundant in the shallow water samples. Shannon diversity index for eukaryotic community structure had a mean of 2.79 and was significantly different across depth but not across stations (*SD* = 0.39, ANCOVA *p*-value for station = 0.416 depth = 6.1 × 10^−10^). In our NMDS analysis, Bray–Curtis distances of relative abundance did have significantly different compositions across depth and IFCB live cell count (ADONIS p for depth = 0.001, live cell = 0.001, detritus = 0.072, station = 0.159, Fig. 3A). The Bray–Curtis distances of relative abundances did have significantly different dispersion across depth and live cell but not across detritus or station (Betadisper ANOVA *p* for depth = 7.0 × 10^−15^, live cell = 4.9 × 10^−15^, detritus = 0.24, station = 0.53).

There were 44 significant genera for high IFCB live cell and 13 significant genera for high IFCB detritus in the two eukaryotic indicator species analyses (Supplementary Table 1 for *p*-values of all indicator species). Unsurprisingly, the dominant eukaryotic genera at 5 m were significant indicators for IFCB live cell level (Fig. 3B); these results are limited in scope as our IFCB live cell level was high only at 5 m. All the genera that were significant indicators for IFCB detritus level were dinoflagellates, most notably *Prorocentrum texanum* and *Bispinodinium angelaceum.*

**Fig. 4:**
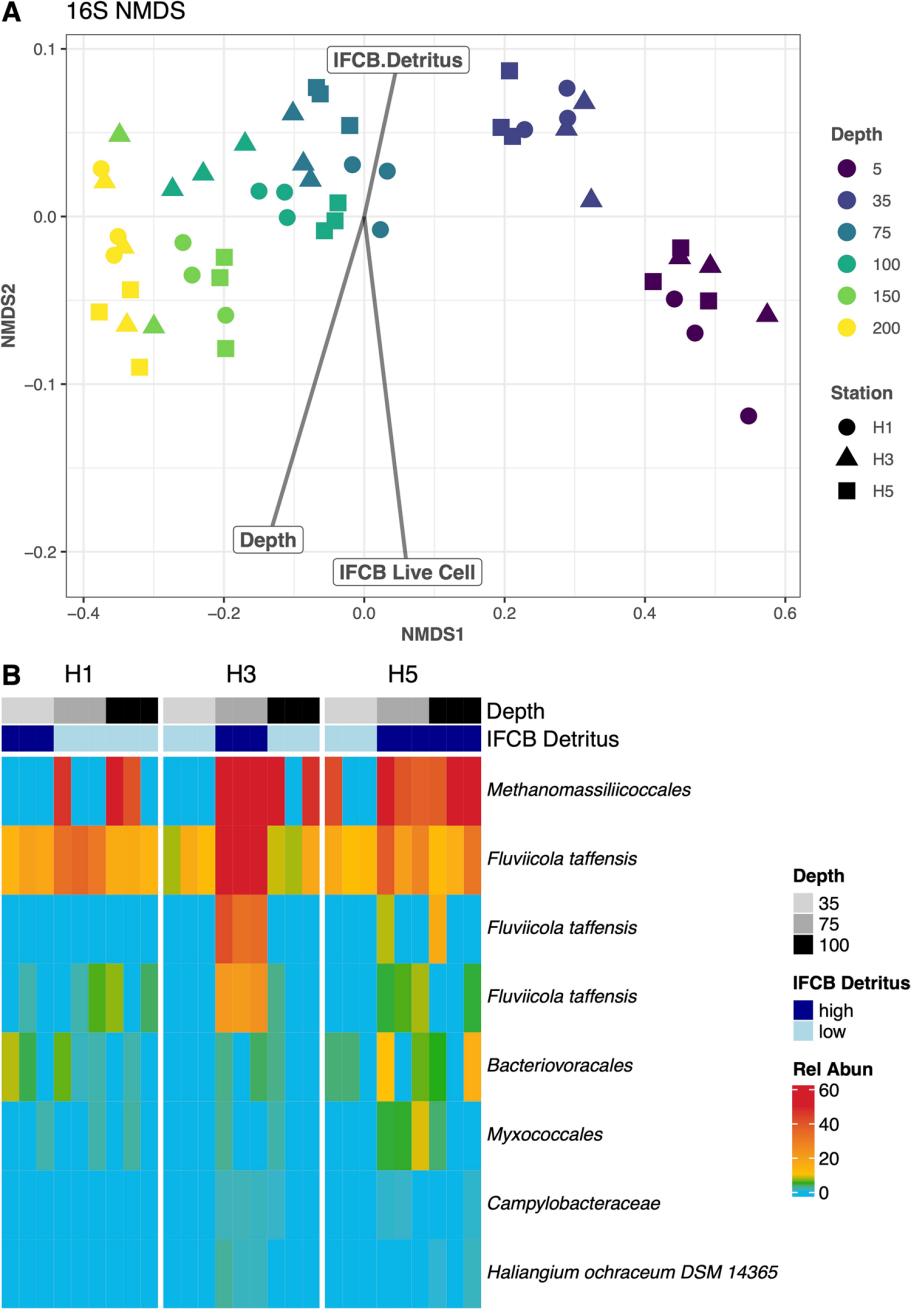
Relative abundance of 16S rRNA gene amplicon sequences across the transect **A** is an NMDS plot (stress = 0.03) where the shape of point is Station (H1, H3 and H5) and color is Depth. IFCB Live Cells and Depth were significant (ADONIS *p* = 0.001) while IFCB Detritus and Station were not significant (ADONIS 0.18 and 0.24, respectively) for the NMDS. **B** Relative abundances of significant indicator species for high (> 1500) IFCB Detritus images at 35, 75 and 100 m depth at each Station (H1, H3, H5)

Prokaryotic community compositions (relative abundance of 16S rRNA sequences, referred as bacterial community composition for simplicity moving forward) were dominated by distinct communities at different depths (Fig. 4A). In the shallow water (5 and 35 m), the genus *Sulfitobacter* dominated relative abundance, while the genera *Nitrosopumilus* and *Thioglobus* were highly relatively abundant in the deeper water (75 to 200 m). Shannon diversity index for bacterial community structure had a mean of 3.19 and was not significantly different across stations but was across depth (*SD* = 0.39, ANCOVA *p*-value for station = 0.70 depth = 6.1 × 10^−12^). In our NMDS analysis, Bray–Curtis distances of relative abundance did have significantly different compositions across depth and IFCB live cell count (ADONIS p for depth = 0.001, live cell = 0.01, detritus = 0.178, station = 0.235, Fig. 4A). The Bray–Curtis distances of relative abundances did have significantly different dispersion across live cell but not across depth, detritus, or station (Betadisper ANOVA p for depth = 0.12, live cell = 0.001, detritus = 0.178, station = 0.73).

In the two bacterial indicator species analyses, there were 22 significant genera for high IFCB live cell and 8 significant genera for high IFCB detritus (Supplementary Table 1 for statistics of all indicator species *p* < 0.05). Just as with the eukaryotes, the dominant bacterial genera at 5 m (including *Sulfitobacter*) were also significant indicators for IFCB live cell level. The genera that were significant indicators for IFCB detritus level included Methanomassiliicoccales and multiple unique amplicon sequence variants that were assigned to *Fluviicola taffensis* (Fig. 4B).

### Metabolic pathway genes and differential abundance analysis

**Fig. 5:**
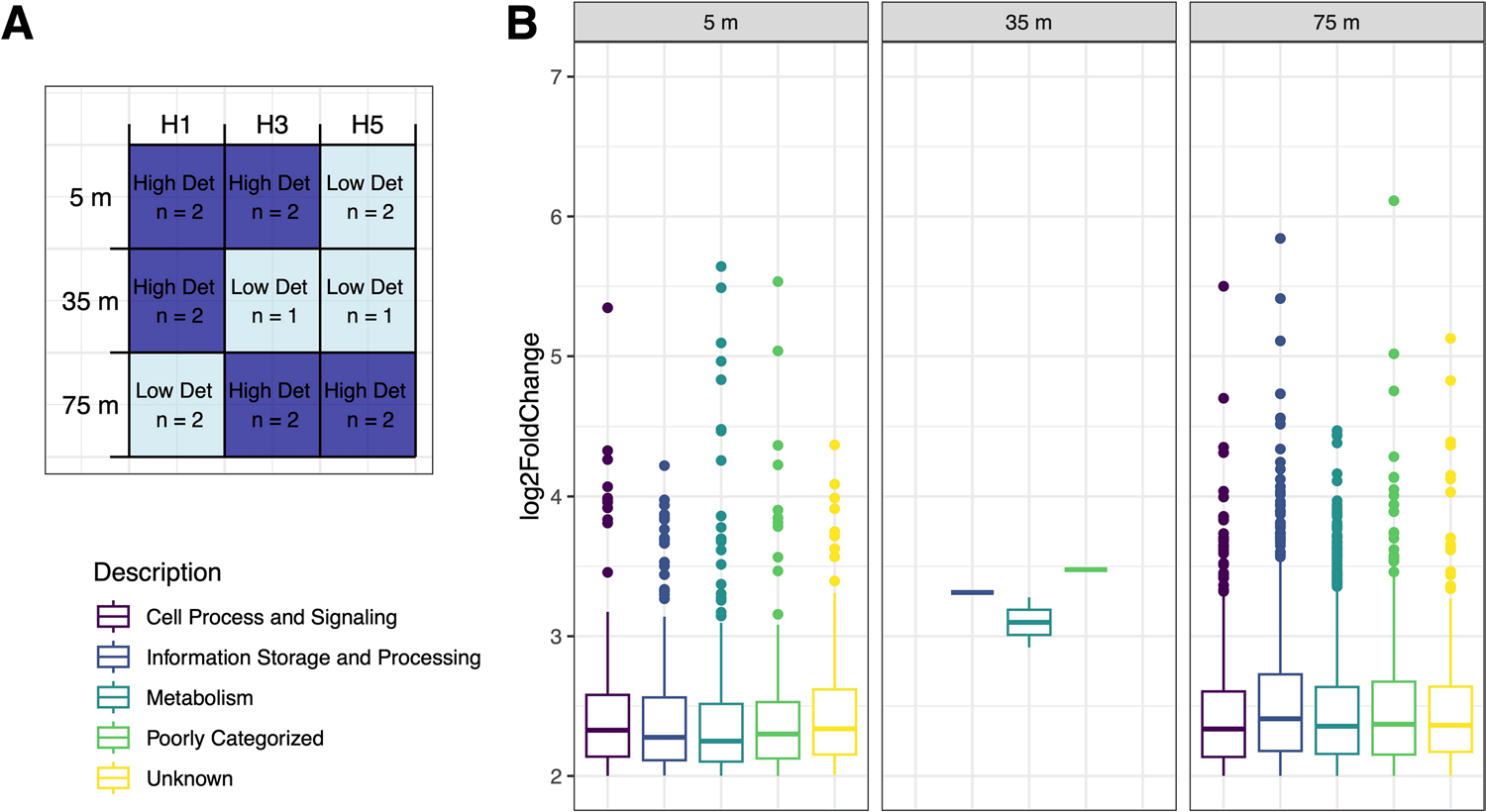
**A** Metagenome samples selected (*n*) for differential abundance analysis for high versus low IFCB Detritus images at each depth, where dark blue color represents samples with high IFCB Detritus levels (> 1500 IFCB images, High Det). **B** All significant (*p* < 0.05) metagenome reads with high log fold change (> 2) from separate differential abundance analyses for high levels of IFCB Detritus conducted at each depth (5 m, 35 m, 75 m), colored by the metagenome read’s COG gene description. The deepest analysis (75 m, including the highest detritus values in Palmer Canyon at H5) resulted in more significantly differentially abundant genes than at the other depths

**Fig. 6:**
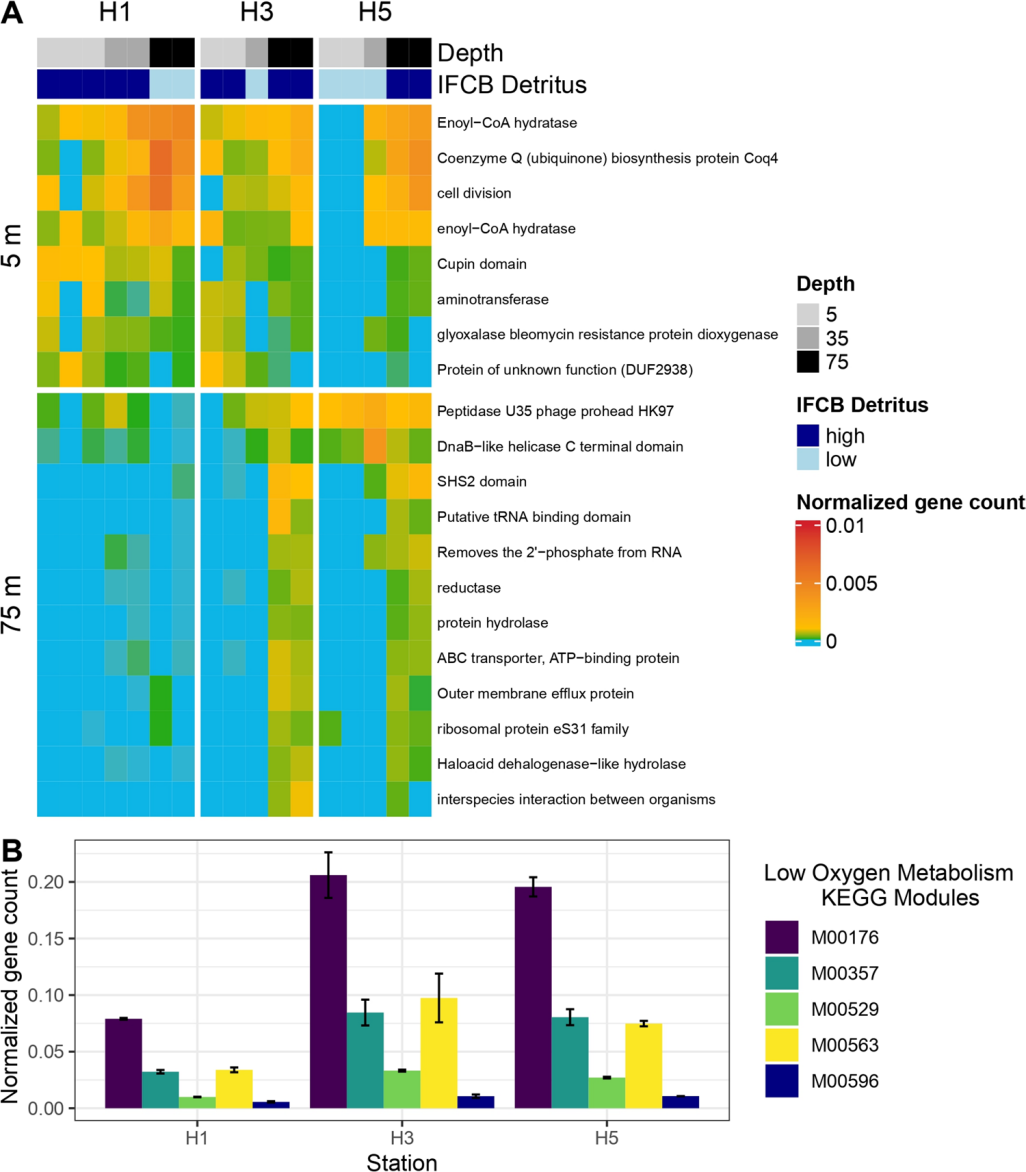
**A** Normalized gene abundance (calculated by dividing relative abundance by the relative abundance of single copy marker gene *rpoB*) of the top twenty significant (*p* < 0.05) metagenome reads with the highest log fold change (> 4.8) from differential abundance analysis for high levels of IFCB Detritus at each depth from either the 5 m or 75 m analyses. **B** Combined normalized gene abundance that mapped to the KEGG modules for low-oxygen metabolism including methanogenesis (M00357 and M00563), sulfate reduction (M00176 and M00596) and denitrification (M00529) at Station H1, H3 and H5 at 75 m

The differential abundance analyses indicated that the high detritus samples at 75 m in Palmer Deep Canyon have a distinct set of metabolic pathway genes, many of which remain unidentified or poorly categorized (Fig. 5). A total of 27,054 metabolic pathway genes were significantly higher abundant in high detritus samples across all the depth-stratified differential abundance analyses (separate 5 m, 35 m and 75 m analysis *p* < 0.05, with exact *p*-values and log fold change values in Supplemental Table 2), with a majority (64%) coming from the differential abundance analysis at 75 m (Fig. 5B). In the 75 m analysis, the twelve most differentially abundant metabolic pathway genes (all with log Fold change > 4.8) had a higher normalized gene count where particle density was high (at H3 and H5 but not at H1, Fig. 6A). Finally in the 75 m analysis, the average (across replicate samples) of the combined normalized abundance of metagenome reads that mapped to the KEGG modules for low-oxygen metabolism including methanogenesis (M00357 and M00563), sulfate reduction (M00176 and M00596), and denitrification (M00529) was almost all significantly higher at Station H3 and H5 (with high IFCB detritus) compared to Station H1 (with low IFCB detritus, ANCOVA *p*-values are M00357 = 1.2 × 10^−3^; M00563 = 1.9 × 10^−2^, M00176 = 3.14 × 10^−4^, M00596 = 1.97 × 10^−3^ and M00529 = 1.7 × 10^−3^, Fig. 6B).

## Discussion

This study provides initial insight into the microbial community composition and metabolic pathway genes in the suspended particle layer of the biological hotspot Palmer Deep Canyon, Antarctica. We identified a layer of living cells near the surface ocean and a suspended detrital particle layer in Palmer Canyon at 75 m. We are confident the detrital material imaged in Palmer Deep is suspended because of previous hydrographic work in the region, including autonomous Slocum glider observations of the Palmer Canyon recirculating eddy and a recent circulation model that demonstrated particle residence times of ~ 175 days within the canyon during the austral summer (Hudson et al. 2019, 2021). The surface layer of living cells and the suspended particle layer had distinct microbial communities, and the detrital layer had differentially highly abundant anerobic metabolic pathway genes including those for sulfate reduction and methanogenesis.

The near surface samples at all three stations (at 5 and 35 m), where the highest living cell abundances were recorded by the IFCB, contained a phytoplankton community composition that is very similar to previous studies. Diatoms such as *Corethron* and *Fragilariopsis* are well understood as the most dominant phytoplankton along the western Antarctica Peninsula (Cimino et al. 2023; Nardelli et al. 2023). In addition to diatoms, dinoflagellates (which includes *Gymnodiniaceae*) and cryptophytes (which includes *Geminigera*) make up over twenty percent of the biovolume of coastal phytoplankton during the peak summer phase close to Palmer Station (Nardelli et al. 2023). A high abundance of the cryptophyte *Geminigera* previously indicated an increasing dominance of small flagellates and cryptophytes at the expense of diatoms with the potential for cascading effects on trophic interactions and carbon export under climate change scenarios (Deppeler and Davidson 2017; Ferreira et al. 2020; Brown et al. 2021). Overall, future study is necessary to determine if the subsurface recirculating eddy has an impact on the retention of surface phytoplankton and the community dynamics that may fuel this biological hotspot.

The near surface bacterial community, especially the dominant genera *Sulfitobacter*, has also been described extensively by previous literature (Luria et al. 2014, 2016; Cimino et al. 2023; Connors et al. 2024). In previous work, the family *Rhodobacteraceae* (the family to which *Sulfitobacter* belongs) was the most abundant during a phytoplankton bloom in the region, and the most dominant during periods of high bacterial activity in a five-year analysis of community structure along the western Antarctic Peninsula (Luria et al. 2016; Bowman et al. 2017). Cultured representatives of *Sulfitobacter* have the ability to breakdown phytoplankton-derived DOM which may explain their dominance during phytoplankton blooms (Choi et al. 2016).

Less is understood about *Nitrosopumilus* and *Candidatus Thioglobus*, the dominant genera at deeper depth regardless of particle density*.* A metagenomic analysis from the region has indicated the average coverages of the genes involved in nitrification, dark carbon fixation, and sulfate reduction are significantly higher with depth (Dutta et al. 2023). *Nitrosopumilus* has been shown to produce oxygen to oxidize ammonia and to fix carbon in a free-living and not particle-attached lifestyle (Kraft et al. 2022). Further study of deeper water particle-attached and free-living microbial communities in polar seas is necessary to better understand these more cryptic biogeochemical cycles, which may include oxygen and nitrogen production.

Our indicator species analysis demonstrated that only dinoflagellates, most notably *Prorocentrum texanum* and *Bispinodinium angelaceum,* were significantly abundant in the particle layer at 75 m within Palmer Canyon. A recent study on a novel *Prorocentrum* identified in the Ross Sea indicated that this new species is unable to photosynthesize under high irradiance, which may explain its dominance in the deeper water column (Bolinesi et al. 2020). The other dinoflagellate identified *Bispinodinium angelaceum* was also initially isolated from a low irradiance site on solid substratum (Yamada et al. 2013). The genera *Prorocentrum* has many species that are mixotrophic phytoplankton with the capacity to ingest prey (Stoecker et al. 2017). Mixotrophy is widespread in polar environments, with mixotrophic plankton persisting during the polar winter when irradiance is low (Stoecker and Lavrentyev 2018). Finally, *Dinophyceae* which includes *Bispinodinium angelaceum* is highly correlated to carbon export globally (Guidi et al. 2016), but the mechanisms underpinning the connection of dinoflagellates to sinking or suspended detrital particles remain largely unexplored. Finally, given that, we used the IFCB to identify detritus as particles with low chlorophyll, dormant or heterotrophic dinoflagellates with low chlorophyll could be a portion of the counted cells.

The bacterial species that were identified by our indicator analysis as significantly highly abundant in the particle layer at 75 m in Palmer Canyon were Methanomassiliicoccales and *Fluviicola taffensis*. *Fluviicola taffensis* belongs to the poorly defined taxonomic group *Cryomorphace,* which in the past has been found in saline waters with enhanced organic loads such as wastewater (Califano et al. 2017; Nilsson et al. 2018; Bowman 2020). Methanomassiliicoccales are hydrogen-dependent methylotrophic methanogenic archaea commonly found in wetlands that contribute to global methane emissions (Weil et al. 2023). Methanogenic archaea have been identified previously in seawater particles and the digestive tract of marine fish and can degrade complex hydrocarbons (van der Maarel et al. 1999; Cheng et al. 2013). A high level of methane production in a suspended particle layer of zooplankton detritus has been observed previously in a lake ecosystem under oxic conditions (Bartosiewicz et al. 2022). The discovery of this anaerobic archaea in the particle layer may indicate that oxygen consumption is not the extent of prokaryotic metabolism on particles in the polar seas.

Our metagenomic sequencing analysis indicated that unique metabolic pathway genes were significantly differentially abundant in the particle layer at 75 m within Palmer Canyon, including anerobic metabolisms for methanogenesis and sulfate reduction. Previous works indicated that low-oxygen microenvironments in suspended aggregates in the oxygenated water column can lead to the presence of anaerobic metabolisms (Simon et al. 2014; Stephens et al. 2024). In our low-oxygen metabolism analysis at 75 m, the most significantly highly abundant pathway found was for assimilatory sulfate reduction, an ubiquitous metabolism that occasionally precedes the metabolic strategy where sulfate is the predominant electron acceptor for the anaerobic oxidation of methane, balancing the production and consumption of methane in marine sediments (Yu et al. 2018; Jespersen and Wagner 2023). This typically niche metabolism may be occurring in the particle layer at 75 m, as the pathways for dissimilatory sulfate reduction and two different methanogenesis pathways (KEGG modules M00357 and M00563) were also significantly higher in the particle layer in our analysis.

A previous study suggests that a symbiotic association of anerobic bacteria within the guts of mid-water protists or krill may be responsible for the anerobic pathways found in our study (Boeuf et al. 2019). We did see evidence for potential facultative anaerobic activity, with significantly higher abundance of the pathway for denitrification (KEGG module M00596) within the particle layer from our low-oxygen metabolism analysis at 75 m. Low but significant rates of denitrification have been measured from experiments on decomposing krill carcasses in the Arctic in previous work, with implications for nitrogen cycling (Franco-Cisterna et al. 2022). Further study is necessary to determine if the differentially abundant anaerobic pathways found in our study are entirely due to anerobic microenvironments within particles derived from decaying phytoplankton, or due to other mid-water environments such as the guts or carcasses of krill and/or protists.

 In this study, we were able to quantify the suspended particle layer of Palmer Deep Canyon, Antarctica, and begin to describe the unique microbial community composition and metabolic potential of these detrital particles. Although the coastal canyons of the western Antarctic Peninsula are well understood as biological hotspots with increased phytoplankton and krill biomass, we know very little about the rate of carbon export and bacterial degradation in the deeper waters of these sites. Our study indicates a mixotrophic community of dinoflagellates and prokaryotic methane metabolism dominate this unique ecosystem. Overall, we provide initial insight of a less explored habitat in the Antarctic environment.

## Supplementary Information

Below is the link to the electronic supplementary material.

Figure 1 A) Autofluorescent (AF) and B) SYBR Stained cell abundances across the triplicate samples at three stations (H1, H3 and H5) with depth. Dot plots (left) demonstrate model populations on the flow cytometer for cell types; for AF this is high chlorophyll (High Chl), low chlorophyll (Low Chl) and high phycoerythrin (High PE) and for SYBR this is high nucleic acid (HNA) and low nucleic acid (LNA). All cell populations decrease with depth at all stations. Supplementary file1 (JPG 3388 KB)

Figure 2 is an NMDS plot (stress = 0.03) of the relative abundance of metagenome reads where the shape of point is Station (H1, H3 and H5) and color is Depth. IFCB Live Cells and Depth were significant (ADONIS p = 0.001 and 0.03, respectively) while Station and IFCB Detritus were not significant (ADONIS 0.19 and 0.15, respectively) for the NMDS.Supplementary file2 (JPG 331 KB)

Table 1 All significant taxa (with stats and p-values) from all four taxa indicator tests. This includes eukaryotic taxa (18S rRNA reads) that were significant indicators for high (> 2000) IFCB Live Cells, eukaryotic taxa (18S rRNA reads) that were significant indicators for high (> 2000) IFCB Detritus, bacterial taxa (16S rRNA reads) that were significant indicators for high (> 2000) IFCB Live Cells, and bacterial taxa (16S rRNA reads) that were significant indicators for high (> 2000) IFCB Detritus. Supplementary file3 (CSV 23 KB)

Table 2 All significant metagenome reads (adj p < 0.05, with log Foldchange reported) from deseq2 analysis from 5 m, 35 m, and 75 m analysis. Supplementary file4 (CSV 11094 KB)

## Data Availability

All sequences available at NCBI SRA BioProject PRJNA901488. Code and data repository for this manuscript is located on the first author’s GitHub.
