# Supplementary figures and images for: Suspended detrital particles support a distinct microbial ecosystem in Palmer Canyon, Antarctica, a coastal biological hotspot
Source: Polar Biol. 2025 Apr 7;48(2):62. doi: 10.1007/s00300-025-03380-y (PMC11976824; doi:10.1007/s00300-025-03380-y)

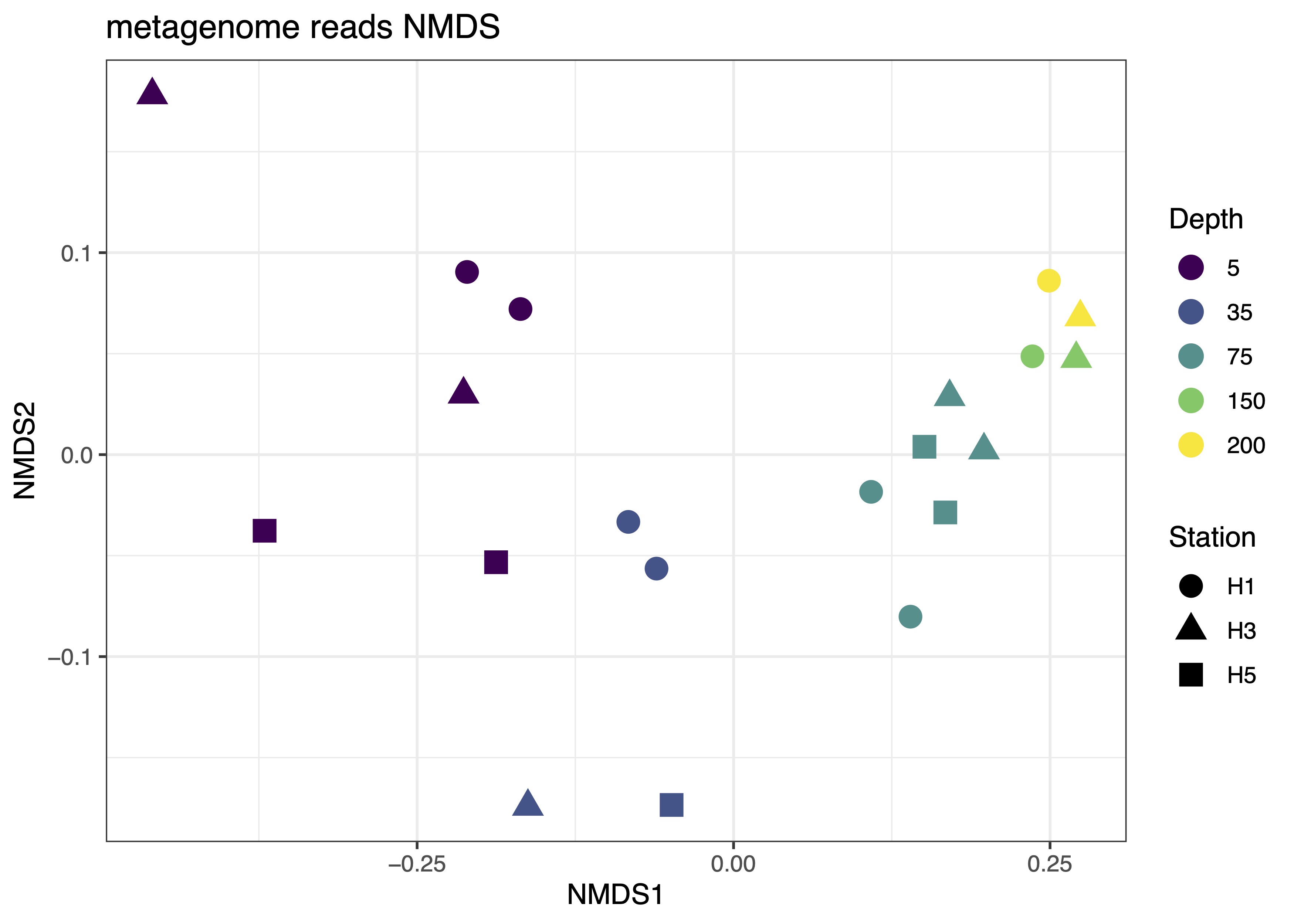

Supplement: Supplementary file 2 — Figure 2 is an NMDS plot (stress = 0.03) of the relative abundance of metagenome reads where the shape of point is Station (H1, H3 and H5) and color is Depth. IFCB Live Cells and Depth were significant (ADONIS p = 0.001 and 0.03, respectively) while Station and IFCB Detritus were not significant (ADONIS 0.19 and 0.15, respectively) for the NMDS.Supplementary file2 (JPG 331 KB) [file 300_2025_3380_MOESM2_ESM.jpg]
